# A case of myocardial infarction caused by obstruction of a drug-eluting stent during the perioperative period

**DOI:** 10.1186/s40981-015-0025-2

**Published:** 2015-12-29

**Authors:** Hiroaki Toyama, Kazutomo Saito, Hiroyuki Anzai, Naoya Kobayashi, Takanori Aihara, Yutaka Ejima, Masanori Yamauchi

**Affiliations:** 1 Department of Anesthesiology, Tohoku University Hospital, 1-1 Seiryomachi, Aoba-ku, Sendai, 980-8574 Japan; 2 Division of Surgical Center and Supply, Sterillization, Tohoku University Hospital, 1-1 Seiryomachi, Aoba-ku, Sendai, 980-8574 Japan; 3 Anesthesiology and Perioperative Medicine, Tohoku University School of Medicine, 2-1 Seiryomachi, Aoba-ku, Sendai, 980-8575 Japan

**Keywords:** Dual antiplatelet therapy (DAPT), Drug-eluting stent (DES), Subacute thrombosis (SAT), Major adverse cardiac event (MACE)

## Abstract

We report a patient who developed drug-eluting stent (DES) thrombosis induced by discontinuation of dual antiplatelet therapy (DAPT) and subsequently had a massive surgical site bleed caused by restarting heparin and DAPT during the perioperative period.

An 85-year-old man visited a local hospital owing to complaints dyspnea. He was diagnosed with laryngeal cancer and was scheduled for a total laryngectomy. Preoperative examinations showed an anteroseptal myocardial infarction. A DES was placed at segment 6 of the coronary artery and DAPT was initiated 27 days before surgery. After admission to our hospital, DAPT was replaced with unfractionated heparin. On the operation day, heparin was discontinued, and a tracheotomy, total laryngectomy and right hemi-thyroidectomy were performed. While recovering from anesthesia, ischemic ST elevation appeared. Cardiac catheterization revealed complete obstruction of the DES by a white thrombus. After recanalization, heparin and DAPT were restarted, and bleeding occurred. The next day, total blood loss was 2755 mL and surgical hemostasis was performed.

Because his serum creatine kinase value was elevated at the cessation of heparin, anticoagulation by unfractionated heparin could not have prevented platelet thrombosis. Therefore, we should performed the tracheostomy to secure the patient’s airway under DAPT or only aspirin therapy a month after the DES implantation, and performed the laryngectomy and right hemi-thyroidectomy five months after the first surgery. This case is serious warnings of perioperative major adverse cardiac events induced by discontinuation of DAPT; unfractionated heparin was an insufficient safeguard against platelet thrombosis, and perioperative massive bleeding induced by restarting antiplatelet and anticoagulation therapy. In addition, a series of human errors, which the cardiologist chosen DES regardless of scheduled total larygectomy, the discontinuation of antiplatelet therapy shortly after a DES placement, and the surgical staffs failed to share the elevated serum CK and CK-MB values, caused life-threatening complications.

## Background

Obstruction of a drug eluting stent (DES) during the perioperative period is a possible and potentially lethal complication of the procedure. To prevent the obstruction of a DES, dual antiplatelet therapy (DAPT), consisting of aspirin and a P2Y12 receptor inhibitor, should be continued for at least a year after DES placement [1]. Therefore, treatment with a bare-metal stent (BMS), which needs DAPT for at least a month [1], and/or plain old balloon angioplasty (POBA) are recommended for patients who have ischemic heart disease and are anticipating non-cardiac surgery [1]. In patients undergoing emergency surgery within a month of DES placement, assessing the risk of bleeding or DES obstruction is difficult. Herein, we report a patient who developed both DES thrombosis and a massive surgical site bleed during the perioperative period.

## Case presentation

**Fig. 1:**
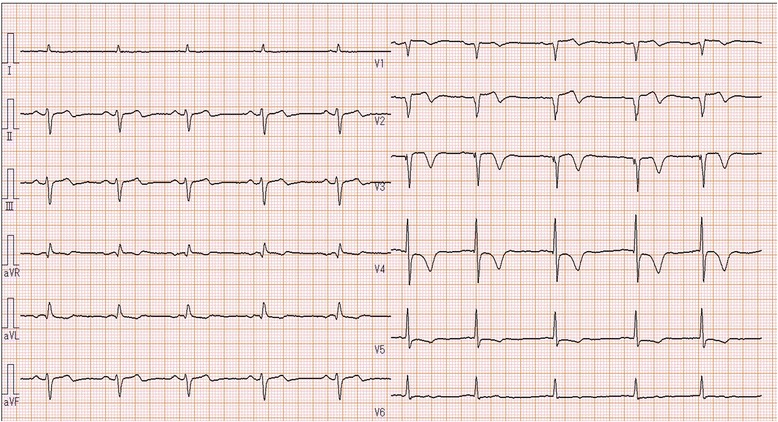
Preoperative 12-lead electrocardiography of the patient, showing slight ischemic ST elevation in leads V1-3 and abnormal ST-T in leads aVL and V2-6

**Fig. 2:**
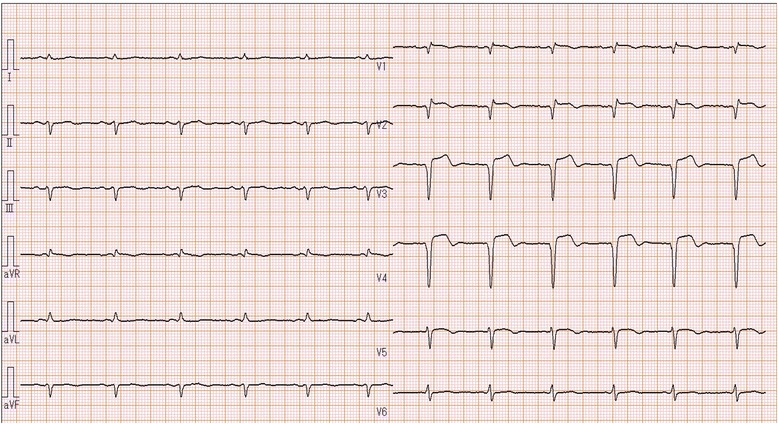
Postoperative 12-lead electrocardiography of the patient, showing ischemic ST elevation in leads V1-5

**Fig. 3:**
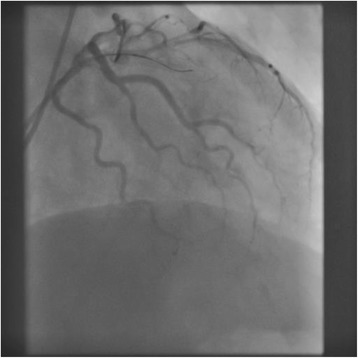
Coronary angiography in the right anterior oblique position after the surgery, showing the left coronary artery and obstruction of segment 6

**Fig. 4:**
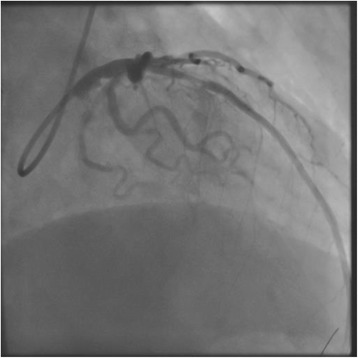
Coronary angiography in the right anterior oblique position during recanalization, showing the obstructed region in the placed drug eluting stent

An 85-year-old man, preserving normal cognitive function, complained of progressive dyspnea and visited a local hospital, where he was diagnosed with laryngeal cancer and scheduled for a total laryngectomy. A preoperative electrocardiogram and echocardiography showed anteroseptal myocardial infarction without symptoms. On 27 days before the laryngectomy, the cardiologist at the local hospital placed a DES at segment 6 (#6) of the anterior descending coronary artery and initiated DAPT, including 100 mg of aspirin and 75 mg of clopidogrel, despite the cardiologist recognized the patient was scheduled laryngectomy. Then, he was admitted to our hospital 10 days before the laryngectomy. Preoperative echocardiography showed anteroseptal hypokinesis and a left ventricular ejection fraction of 36 %. A 12-lead electrocardiogram showed a slight ischemic ST elevation in leads V1-3 and an abnormal ST-T in leads aVL and V2-6 (Fig. 1). Cardiologists at our hospital assessed that the patient’s myocardium perfused by #6 had no function, and obstruction of the DES would have little effect on cardiac function from the echocardiographic finding. The cardiologists started 400 U/h of unfractionated heparin as a substitute for DAPT 6 days before the laryngectomy. Five days before the laryngectomy, we were consulted about the patient and warned the surgeons about the risks associated with discontinuation of DAPT. Six hours before the laryngectomy, heparin was discontinued. The surgeons checked the serum creatine kinase (CK), CK-MB and activated partial thromboplastin time (aPTT) values at the cardiologist’s direction, three hours before the laryngectomy. An hour before the laryngectomy, a central clinical laboratory staff noticed the abnormal values of the CK and CK-MB (Table 1), which were reported to one of the surgeons by phone. The reported surgeon failed to inform us about the abnormal values. The patient entered the operating room in a wheelchair. He showed no significant changes in 3-lead electrocardiogram and did not complain of a chest pain. Oxygenation and 3 mg/h of nicorandil were started, and an arterial line was placed. Then, tracheotomy was performed under regional anesthesia with supplementation of fentanyl. After the tracheal intubation via tracheostomy, general anesthesia was induced and maintained with propofol (2.5–1.3 μg/mL), remifentanil (0.33–0.13 μg/kg/min), fentanyl (total: 0.3 mg) and rocuronium. Circulation was supported by continuous nicorandil and phenylephrine, and bolus ephedrine. A total laryngectomy and right hemi-thyroidectomy were performed. Duration of the surgery was 211 min, and blood loss during surgery was 7 mL. Little change was observed in his 3-lead electrocardiogram during surgery. After the completion of surgery, anesthetic agents were discontinued. During recovery from anesthesia, the patient’s heart rate and arterial pressure elevated to 100/min and 190/80 mmHg, respectively, and 0.5 mV of ischemic ST elevation was confirmed in lead II of the electrocardiogram. Therefore, the intravenous nicorandil was increased to 6 mg/h and bolus nicardipine (0.4 mg) and landiolol (10 mg) were administered for reduction of blood pressure and heart rate. The 12-lead electrocardiogram showed an ischemic ST elevation in leads V1-5 (Fig. 2), and the patient complained of chest pain with gestures after recovery. A bolus of 10 mg of morphine and continuous 1 μg/kg/min of isosorbide dinitrate were administered and we quickly called the cardiologist, who immediately decided to perform percutaneous coronary intervention. The patient was transferred to the cardiac catheterization laboratory under sedation by dexmedetomidine (0.4 μg/kg/h). We regretted to find the elevated CK and CK-MB values at the time. At the laboratory, complete obstruction of the DES was observed (Fig. 3), and aspiration of the white thrombus, caused by a subacute thrombosis (SAT), was performed. After recanalization of the DES (Fig. 4), the patient’s chest pain and the ST elevation in the electrocardiogram disappeared, and 400 U/h of unfractionated heparin was restarted. After that, the patient was transferred to the intensive care unit (ICU), where 100 mg of aspirin and 3.75 mg of prasugrel were administered through a feeding tube. Then, bleeding from the surgical site began and the patient’s hemodynamic status gradually deteriorated. Therefore, heparin, aspirin and prasugrel were stopped. On the morning of the 1st postoperative day (POD), the patient’s aPTT was still markedly prolonged (Table 1), and the total blood loss reached 2755 mL. In addition, 1120 mL of red blood cells (RBCs) and 480 mL of fresh frozen plasma (FFP) were transfused. Thereby, surgical hemostasis and transfusion of RBCs (920 mL) and FFP (480 mL) were performed. Aspirin and prasugrel were restarted on the 3rd POD. Hemorrhage and re-occlusion of DES did not reoccur, and the patient was discharged on foot on the 34th POD.

**Table 1:** Serum creatine kinase, creatine kinase MB, and activated partial thromboplastin time values

	7 days before surgery	The day of surgery		1 POD	2 POD	5 POD	6 POD
		Morning	Evening				
CK (U/L)	84	1345	2485	1690	980	212	164
CK-MB (U/L)	8	175	364	247	59	11	10
aPTT (s)	32.8	44.8	-	94.2	44.6	-	-

*POD* postoperative day, *CK* creatine kinase, *CK-MB* creatine kinase MB, *aPTT* activated partial thromboplastin time

## Discussion

2014 ACC/AHA guideline recommend that, for patients who require PCI but are scheduled for elective non-cardiac surgery in the subsequent 12 months, balloon angioplasty or BMS implantation followed by 4 to 6 weeks of DAPT are reasonable strategies [1]. For patients with DES who must undergo urgent surgical procedures that mandate the discontinuation of DAPT, however, it is reasonable to continue aspirin if possible and restart the P2Y12 inhibitor as soon as possible in the immediate postoperative period [1, 2]. And the study showed that incidence of major adverse cardiac event (MACE) was significantly higher (10–15 %) during the first 30 days after the stent implantation [3]. In our case, placement of a DES was the primary issue for the patient, who urgently required tracheostomy because of airway narrowing. The discontinuation of antiplatelet therapy shortly after the myocardial infarction and DES placement induced the SAT, which was the secondary issue.

Bridging with anticoagulants, such as low-molecular-weight heparin, after interruption of DAPT during the perioperative period is advised [2]. In Japan, unfractionated heparin, having no evidence and used empirically, is also advised [4]. But the newer perioperative guideline recommend continue DAPT in patients undergoing urgent noncardiac surgery during the first 4 to 6 weeks after BMS or DES implantation unless the risk of bleeding outweights the benefit of stent thrombosis prevention [1]. In addition, there has not been any evidence demonstrating the efficacy of bridging with anticoagulants using heparin. In this case, we performed anticoagulation therapy using unfractionated heparin. Nevertheless, the serum CK-MB value of the patient was elevated at the time of cessation of heparin despite a slightly prolonged aPTT (Table 1). The all surgical staffs failed to share the elevated serum CK and CK-MB values at that point and failed to cancel the surgery, which were the third issue. And a white thrombus in the DES was confirmed at the time of recanalization. Although his preoperative aPTT was not markedly prolonged, anticoagulant therapy of 400 U/h of unfractionated heparin could not have prevented platelet thrombosis in this case.

It has been reported that the cumulative incidences of bleeding 30 days after surgical procedures were significantly higher in patients with DAPT than in patients with single or no antiplatelet therapy [5]. In this case, the first surgery was performed after discontinuation of both antiplatelet and anticoagulation therapy. Therefore, surgical hemostasis was easily obtained and not strictly performed, and bleeding only became obvious after restarting heparin and DAPT. If the surgery was performed under continuous DAPT, however, similar or massive bleeding might have occurred in the perioperative period. Because the patient had been placed a DES, we should have first performed the tracheostomy on the patient with strict hemostasis to secure the airway under continuation of DAPT or only aspirin a month after the DES implantation. Then, six months after the DES implantation, we should have performed the laryngectomy and right hemi-thyroidectomy [1].

Because the patient might have mild impairment in expression of chest pain due to progressive laryngeal cancer and/or feel little pain due to nerve damage by old myocardial infarction, the patient could complained less chest pain at the time of visiting to local hospital and entering the operation room, which could induced misjudgment. And a series of human errors occurred and caused life-threatening complications. Human errors were as follows: the cardiologist at the local hospital chosen DES instead of BMS or POBA regardless of scheduled total larygectomy; the root cause of this incident, the discontinuation of antiplatelet therapy shortly after the myocardial infarction and DES placement, and the all surgical staffs failed to share the elevated serum CK and CK-MB values and failed to cancel the surgery. After this case, we proposed to create the hospital task force for updating and promoting the perioperative PCI, antiplatelet and anticoagulation procedures. This task force consisted of the representatives from departments of all surgery, anesthesiology, cardiology, gastroenterology, pharmacy and hospital medical safety management office. Then, this task force announced the updated those procedures in our hospital. And now we are exploring a method of enhancing the detection of laboratory test abnormality shortly before the surgery.

## Conclusion

Because the patient had airway stenosis with a recent placement of DES, we should have first performed the tracheostomy on the patient with strict hemostasis to secure the airway under continuation of DAPT or only aspirin a month after the DES implantation. Then, six months after the DES implantation, we should have performed the laryngectomy and right hemi-thyroidectomy. This case is serious warnings of perioperative MACE induced by discontinuation of DAPT; 400 U/h of unfractionated heparin was an insufficient safeguard against platelet thrombosis, and perioperative massive bleeding then induced by restarting antiplatelet and anticoagulation therapy. In addition, a series of human errors, which the cardiologist at local hospital chosen DES regardless of scheduled total larygectomy, the discontinuation of antiplatelet therapy, and the surgical staffs failed to share the elevated serum CK and CK-MB values, caused life-threatening complications. Anesthesiologists should promote the information about the perioperative risks and managements of PCI and DAPT to surgeons and cardiologists further widely.

## Consent

Written informed consent was obtained from the patient for publication of this Case report and any accompanying images. A copy of the written consent is available for review by the Editor-in-Chief of this journal.

## Abbreviations

#6: segment 6
aPTT: activated partial thromboplastin time
BMS: bare-metal stent
CK: creatine kinase
DAPT: dual antiplatelet therapy
DES: drug eluting stent
FFP: fresh frozen plasma
ICU: intensive care unit
MACE: major adverse cardiac events
POBA: plain old balloon angioplasty
POD: postoperative day
RBCs: red blood cells
SAT: subacute thrombosis
